# 3D-nanoarchitectured Pd/Ni catalysts prepared by atomic layer deposition for the electrooxidation of formic acid

**DOI:** 10.3762/bjnano.5.16

**Published:** 2014-02-12

**Authors:** Loïc Assaud, Evans Monyoncho, Kristina Pitzschel, Anis Allagui, Matthieu Petit, Margrit Hanbücken, Elena A Baranova, Lionel Santinacci

**Affiliations:** 1 Aix-Marseille Université, CNRS, CINaM UMR 7325, 13288, Marseille, France; 2 Department of Chemical and Biological Engineering, Center for Catalysis Research and Innovation, University of Ottawa, 161 Louis-Pasteur St., Ottawa, ON, K1N 6N5, Canada

**Keywords:** anodic aluminum oxide, atomic layer deposition (ALD), direct formic acid fuel cells, electrooxidation, nanostructured catalysts, Pd/Ni

## Abstract

Three-dimensionally (3D) nanoarchitectured palladium/nickel (Pd/Ni) catalysts, which were prepared by atomic layer deposition (ALD) on high-aspect-ratio nanoporous alumina templates are investigated with regard to the electrooxidation of formic acid in an acidic medium (0.5 M H_2_SO_4_). Both deposition processes, Ni and Pd, with various mass content ratios have been continuously monitored by using a quartz crystal microbalance. The morphology of the Pd/Ni systems has been studied by electron microscopy and shows a homogeneous deposition of granularly structured Pd onto the Ni substrate. X-ray diffraction analysis performed on Ni and NiO substrates revealed an amorphous structure, while the Pd coating crystallized into a fcc lattice with a preferential orientation along the [220]-direction. Surface chemistry analysis by X-ray photoelectron spectroscopy showed both metallic and oxide contributions for the Ni and Pd deposits. Cyclic voltammetry of the Pd/Ni nanocatalysts revealed that the electrooxidation of HCOOH proceeds through the direct dehydrogenation mechanism with the formation of active intermediates. High catalytic activities are measured for low masses of Pd coatings that were generated by a low number of ALD cycles, probably because of the cluster size effect, electronic interactions between Pd and Ni, or diffusion effects.

## Introduction

**Scheme 1:**
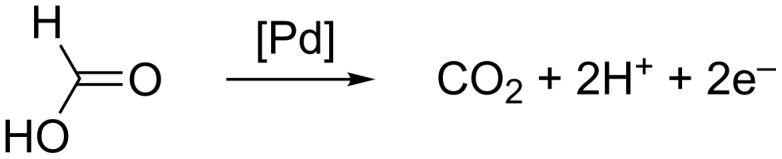
Pd-catalyzed electrooxidation of HCOOH on Pd surfaces.

Over the last decade, the miniaturization of fuel cells for the fast expanding market of portable devices has become a challenging research topic. Direct formic acid fuel cell (DFAFC) systems as electrochemical power sources have many advantages such as the low-toxicity, unlike methanol, the low cost, and the low fuel crossover at a high power density [1–3]. Palladium is a good candidate to catalyze the electrooxidation of formic acid thanks to its good stability at low pH and its high activity [4–8]. The electrooxidation of HCOOH on Pd results in the formation of CO_2_ and protons [7], which is a direct dehydrogenation pathway through the formation of active intermediates without the generation of poisonous CO species (Scheme 1).

Despite the advantages of Pd catalysts for the electrooxidation of formic acid, the activity is still not satisfactory enough for commercial applications and more importantly, Pd tends to dissolve and deactivate quickly by the impurities present in the electrolyte [9]. Additionally, reducing the noble metal loading by alloying Pd with a second cheap transition metal is essential for a viable development of DFAFCs. In recent studies, several metals such as Cu, Ni, Fe or Pt alloyed with Pd have been tested [10–13] for the electrooxidation reaction of HCOOH, and have shown a significant increase of the catalytic activity when compared to pure Pd. Amongst them, the Pd/Ni bimetallic system has shown very promising results due to the favorable electronic effects that Ni brings into the system.

It is also well-known that decreasing the size of the active particles and thus increasing the electro-active surface area of the catalyst are interesting ways to improve the electrooxidation of HCOOH. Nanostructured substrates such as nanowires, nanorods, nanopores or nanotubes have thus been investigated to enhance the catalytic efficiency and to reduce the costs [14]. On the other hand, the physical, chemical and electrochemical properties of the nanostructures are highly correlated with the technique of fabrication. Among the numerous methods that have been recently explored, the use of atomic layer deposition (ALD) to fabricate and/or functionalize nanostructures appears to be very promising. Catalysts grown by ALD often demonstrated similar or enhanced properties as compared to those grown by conventional methods, such as impregnation, ion-exchange, and deposition–precipitation [15–16]. ALD has initially been used to produce oxide layers to support the catalysts [17], but two additional approaches have been recently proposed: ALD is either used to grow metallic clusters or it is applied to protect those metallic clusters with an ultrathin metal oxide layer (see, e.g., the reviews [18–20]). This deposition method is particularly interesting for electrocatalysis because it allows an accurate control of both growth rate and composition of the catalyst, and it provides a high coverage of high aspect ratio nanostructures [21–23]. It is therefore possible to precisely design catalysts onto nanoarchitectured supports that exhibit enhanced abilities for fuel cell applications [24–25].

**Figure 1:**
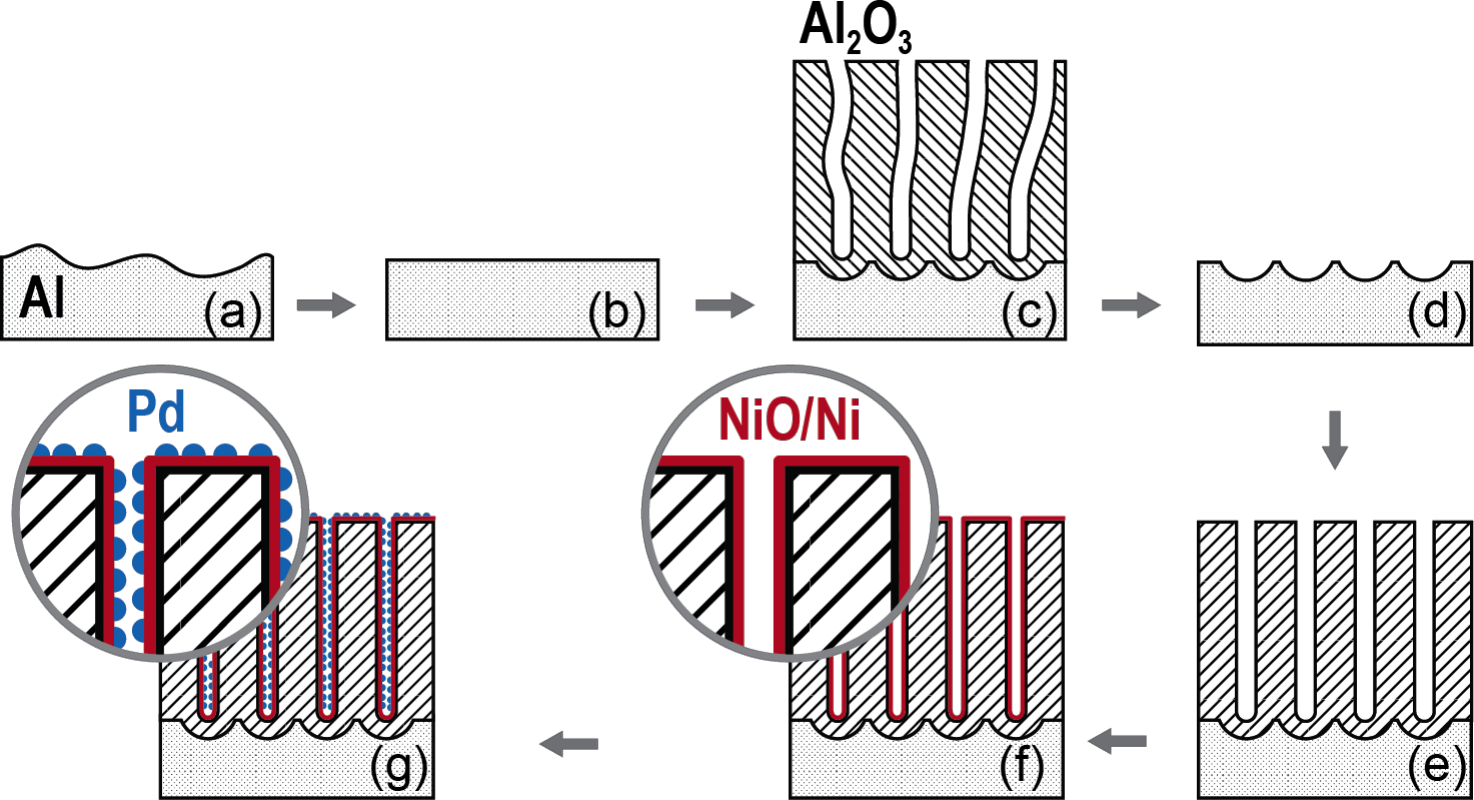
Schematic description of the anodic alumina template fabrication and successive functionalization. (a) Initial Al disc, (b) electropolished Al disc, (c) anodic Al_2_O_3_ porous layer, (d) pre-ordered Al disc after removal of the AAO sacrificial layer, (e) self-ordered AAO membrane, (f) Ni/NiO film deposited by ALD and reduced by annealing post treatment, (g) Pd cluster layer grown by ALD.

As previously proposed [26], nanoporous anodic aluminum oxide (AAO) has been used as nanostructured support for the Pd catalysts. The AAO membranes are attractive because they exhibit a high specific surface area and the pore diameter and length can be tailored easily [27–28]. In this study, the usual two-step anodization process shown in Figure 1a–e, has been used to grow well-ordered porous structures. Ni and Pd are then successively deposited into the templates by ALD. The alumina membranes are firstly coated by NiO that is reduced to metallic Ni by annealing under H_2_ atmosphere [29–30] (Figure 1f). The Pd clusters are then deposited directly onto the Ni films (Figure 1g). Both NiO and Pd deposition processes have been monitored by quartz crystal microbalance (QCM). The morphology, the chemical composition and the crystalline structures have been investigated by scanning and transmission electron microscopy (SEM and TEM) and atomic force microscopy (AFM), X-ray photoelectron spectroscopy (XPS) and X-ray diffraction (XRD), respectively. The electrocatalytic activity of the Pd/Ni systems, which were deposited on three-dimensional alumina membranes with various mass content ratios, for the electrooxidation of formic acid in acidic solution has been studied by cyclic voltammetry (CV).

## Results and Discussion

### Nickel deposition

**Figure 2:**
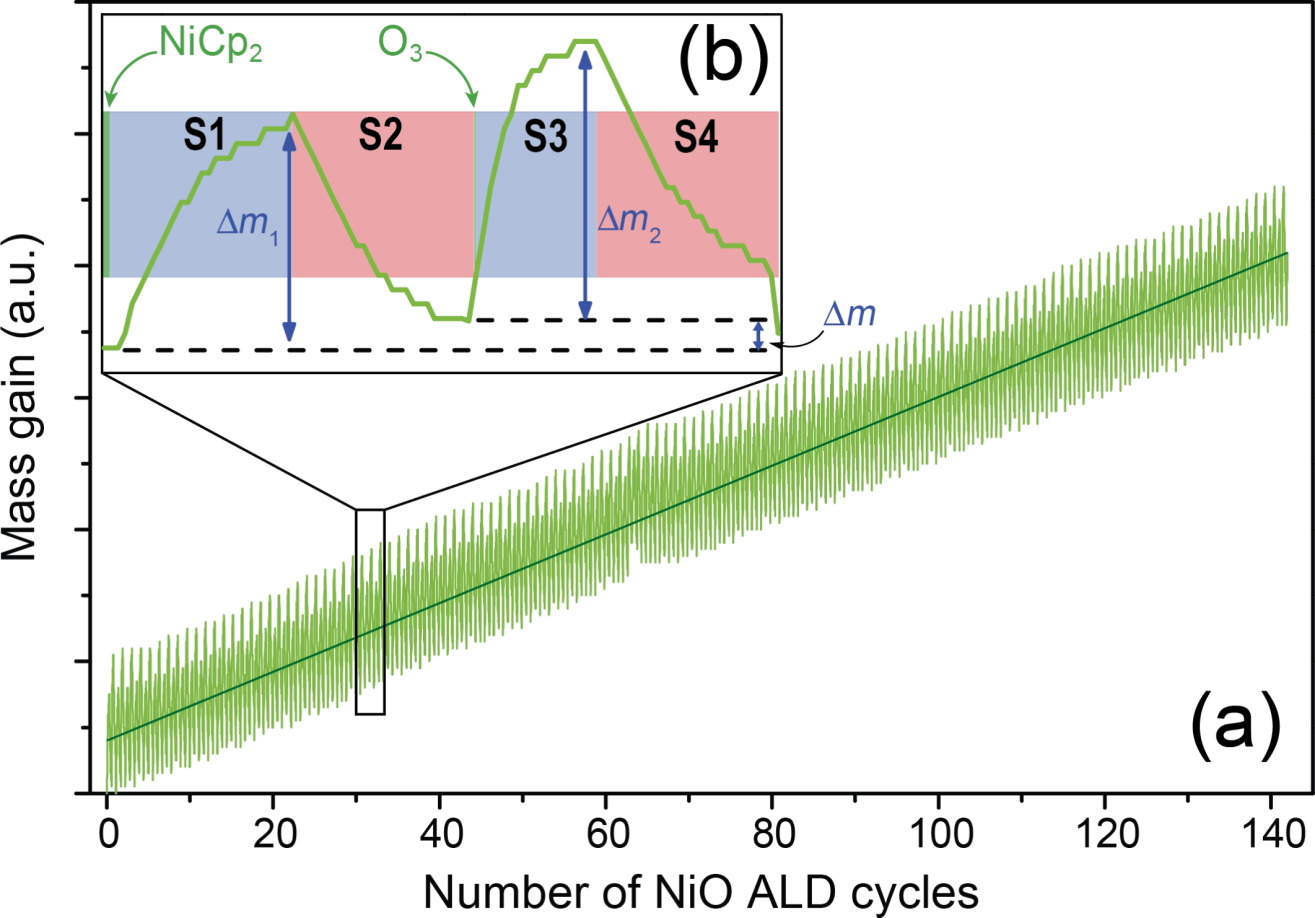
(a) In situ QCM measurement of the NiO mass gain during the ALD process. (b) Enlarged view of the mass gain for one ALD cycle. Δ*m* corresponds to the net mass increase after the ALD cycle.

Since ALD processes have been developed mainly for metal oxide and nitride thin films, metal depositions have been hampered mostly by the lack of relevant and stable precursors [31]. Although a new class of precursors that facilitates the direct metal deposition, has recently been proposed [32], several metals are often grown through a two-step process: (i) deposition of the metallic oxide and (ii) subsequent reduction (see, e.g., [29–30]). Metallic Ni is therefore grown by using such an approach [33–34]: the deposition of NiO is carried out from nickelocene (NiCp_2_) and O_3_ precursors and the reduction of this oxide film to metallic Ni is obtained by a reductive annealing process under H_2_ atmosphere. The relative mass, *m*, gain and loss have been monitored during the process by QCM and are plotted in Figure 2a. A regular cyclic variation of the mass vs the number of ALD cycles is observed with an overall linear evolution, which is typical for an ALD process with constant growth rate. An enlarged view of one cycle presented in Figure 2b shows in detail the process during the four successive steps of the NiO ALD sequence. The QCM measurements indicate that the exposure and purging duration are optimized for both NiCp_2_ and O_3_ pulses. The mass variations are indeed reaching a plateau at the end of the exposure and purging stages. After the short NiCp_2_ pulse (green period on the far left of Figure 2b), the mass increases progressively up to a maximum (Δ*m*_1_) during the exposure phase (S1). Then a mass loss is measured during the purging phase (S2). A similar trend is observed after the O_3_ pulse: a mass increase (Δ*m*_2_) is measured during the exposure time (S3) followed by a total mass loss during the purging phase (S4).

It is difficult to correlate the mass gain and loss measured by the QCM with a reaction mechanism. Thus few data can be found in literature about such chemical processes. However, Martinson et al. proposed a detailed investigation of the Fe_2_O_3_ formation from FeCp_2_ and O_3_ precursors by using quadrupole mass spectrometry (QMS) [35]. Since the precursors used for this deposition are close to those employed in the present study, the Martinson mechanism may be adapted to the deposition of NiO using NiCp_2_ and O_3_. Therefore, the S1 period could be ascribed to the adsorption of NiCp_2_ on the whole surface, which after reaction on the active sites yields a –NiCp group on the surface and one cyclopentadiene molecule is released. Note that Martinson et al. have also detected cyclopentadione as a byproduct in the ferrocene process. During the S2 stage, the desorption of the precursors that have not reacted with active surface sites seems to occur. The net mass gain detected after the nickelocene pulse, exposure and purging could be attributed to the bonding of a –NiCp group with a surface –OH group.

According to the study performed on Fe_2_O_3_ [35], the S3 and S4 stages could be associated to a combustion of the chemisorbed –NiCp groups. Those cyclopentadienyl groups should therefore be cracked with the production of CO_2_ and H_2_O. The surface is then activated again with hydroxyl functions onto the Ni atoms. While a mass loss, corresponding to the combustion of Cp, is expected after the O_3_ exposure, the QCM measurements (Figure 2b) do not show any net mass decrease during the step. This unexpected measurement could be attributed to a cooling effect of the vector gas (Ar) on the quartz. The QCM is indeed a very sensitive characterization tool as the sensor oscillation frequency can easily change when low temperature variations occur. Additional experiments have thus been performed by pulsing only ozone. Without NiCp_2_, the QCM data exhibit a low level background and no regular increase. This indicates that the general trends of *m* vs *t* shown in Figure 2a are relevant but no mechanistic information can be deduced from the detailed interpretation of the QCM measurements. QMS investigation would be required to support the proposed chemical mechanism.

**Figure 3:**
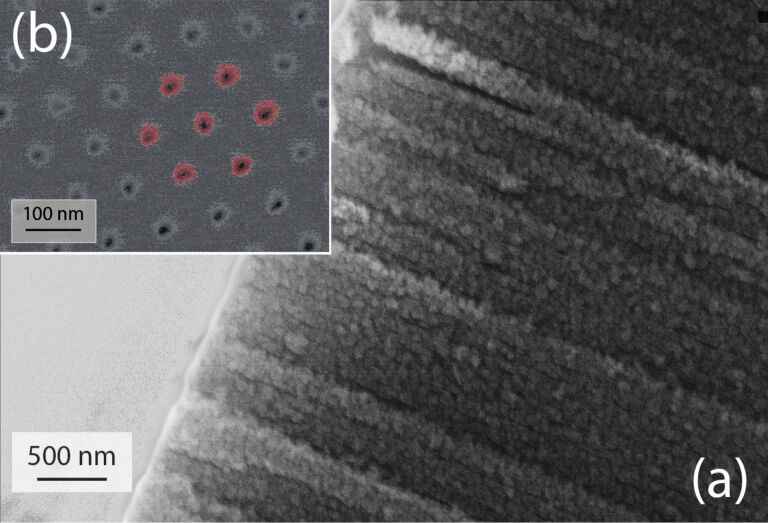
(a) SEM cross section of a NiO layer deposited in AAO membrane. (b) SEM image (obtained in backscattering electron mode) showing NiO grown by ALD within the AAO template. The NiO top layer has been removed by a short Ar sputtering in order to reveal the NiO film coating the vertical pore walls. The NiO deposit is emphasized on the picture using a red overlay.

**Figure 4:**
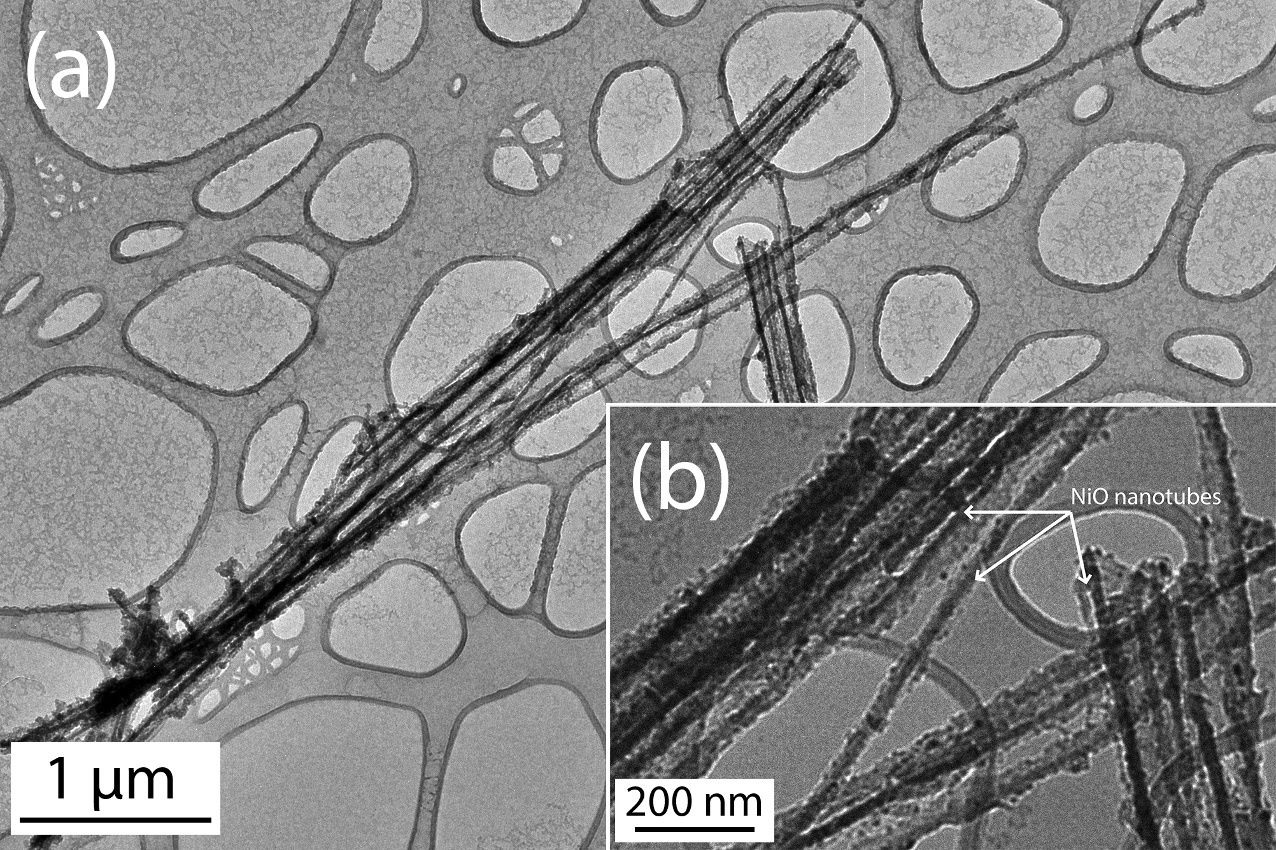
(a) TEM image of NiO nanotubes after alumina template removal. (b) Enlarged view of NiO nanotubes.

In order to characterize their morphology, the resulting NiO/Ni layers have been studied by electron microscopy. The backscattering electron detection mode was used to enhance the chemical contrast of the image shown in Figure 3. The NiO deposit (red color in the figure) is clearly visible within the Al_2_O_3_ pores. The NiO film is approximately 10 nm thick after 1000 ALD cycles. The TEM picture presented in Figure 4 shows the as-grown NiO layer deposited within the AAO membrane after removal of the alumina template. The average length of the nanotubes is 5 μm, which indicates that the exposure time to NiCp_2_ is sufficiently long to allow for the deposition to proceed deeply on the entire surface of the pores. On such a TEM image, several NiO nanotubes can be observed. The NiO layer covers the AAO template homogeneously. Note that no gradient of NiO loading is observed in the deep section of the template. The quantity of matter is identical at the top and the at the bottom of the pores. This is attributed to the self-limiting process of the ALD. The tuning of the duration of the surface exposition to the precursors allows for the reaction of the molecules with the activated surface of the three-dimensional substrates, which exhibit a high aspect-ratio geometry.

**Figure 5:**
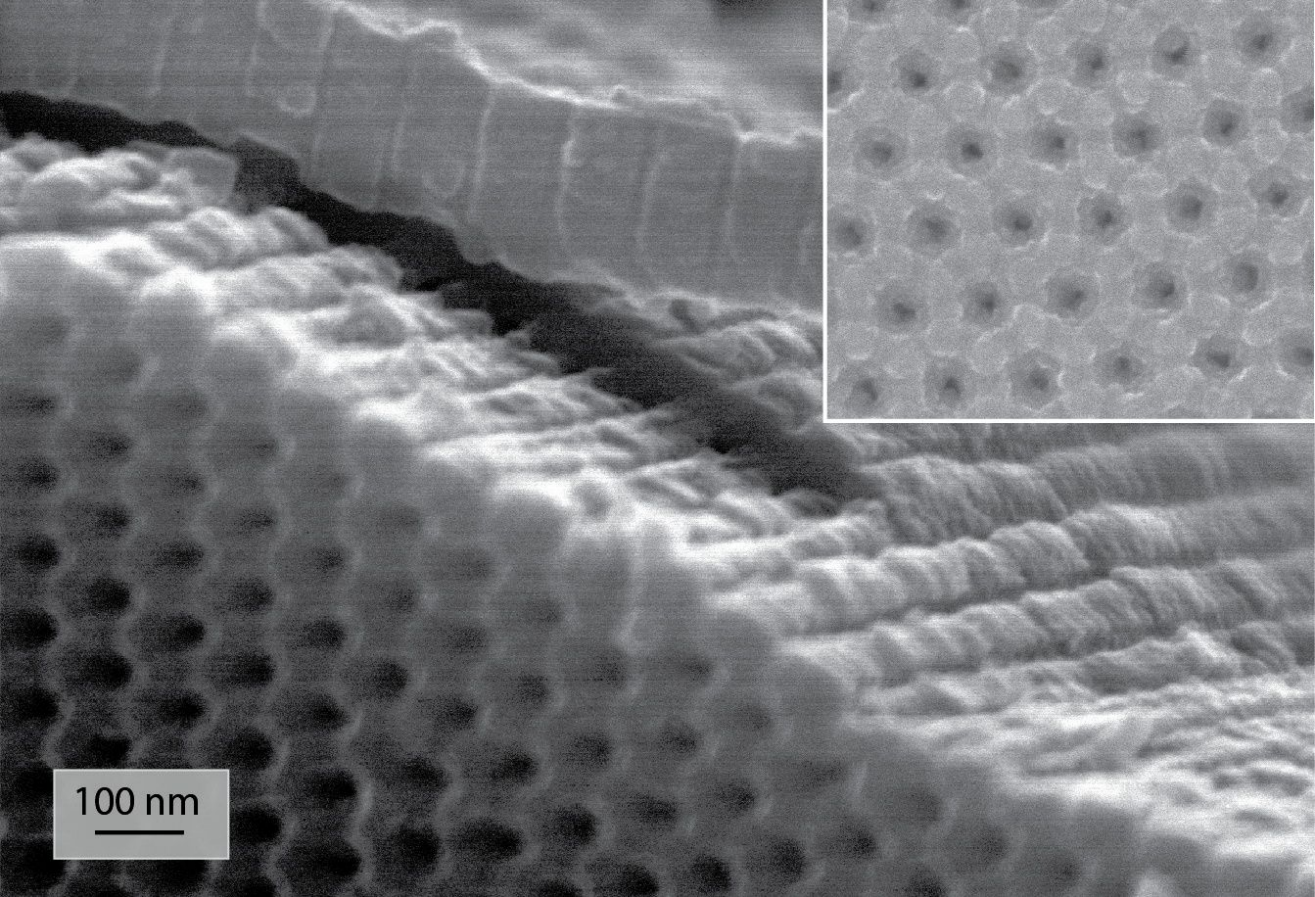
SEM image of Ni layer deposited in an AAO template after 3 h annealing in H_2_ at 300 °C of the initially deposited NiO by ALD. The inset shows a detailed top-view of the surface.

The TEM image shows that the morphology of the NiO deposit is highly granular. It therefore increases the active surface area of the electrode for a higher interaction with the electrolyte. Note that the NiO layer is an efficient barrier between the solution and the AAO since we have never observed the dissolution of the AAO during the electrochemical characterizations. To get a metallic Ni film, the as-grown NiO deposit has been annealed in H_2_ atmosphere at 300 °C. The SEM observations indicate no significant morphological modifications of the Ni after the reductive annealing (Figure 5). The NiO film shown in Figure 3 (before annealing) exhibits a granularity slightly higher than in Figure 5 (after annealing). It is however difficult to get quantitative results from such SEM pictures. Note that inversely, a treatment performed in Ar at higher temperature (*T* = 700 °C) has shown a strong increase of the granularity after such annealing [34].

**Figure 6:**
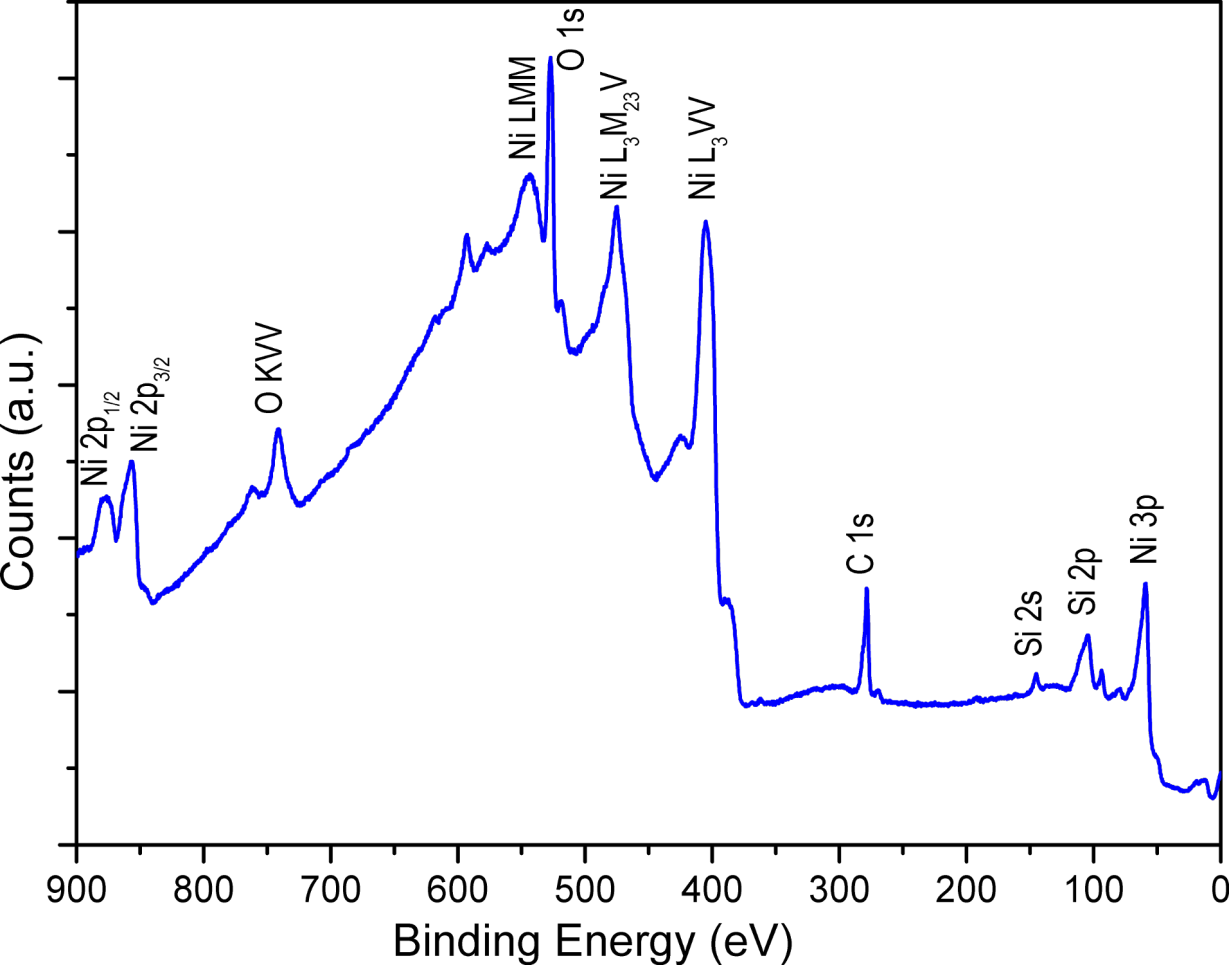
XPS survey spectrum of metallic Ni.

The chemical composition as well as the crystal structure of the NiO/Ni layer have been analyzed before and after the reduction stage. The XRD analyses performed on as-grown NiO and after the reduction process indicate that both NiO and Ni layers deposited on AAO are amorphous (XRD patterns are shown in Figure S1, Supporting Information File 1). The surface chemistry of the sample after the reductive annealing of the Ni deposit has been analyzed by XPS. As expected, the spectrum shown in Figure 6 exhibits peaks corresponding to Ni, C and Si but also to O. Although the Ni 2p, Ni 3p and Auger peaks indicate the presence of metallic Ni, the O 1s peak suggests that the Ni deposit remains partially oxidized after the reductive treatment. Since the XPS analysis provides information on the outermost surface, the Ni–O contribution can either originate from an only partial reduction of the initial NiO layer or from the oxidation of the sample while transferring it to the XPS chamber. Note that a contribution coming from SiO_2_ in the XPS spectrum in the O 1s binding energies region is possible since an interfacial SiO_2_ layer is formed between Si and NiO (Figure S2, Supporting Information File 1). However, the contribution of Si in the survey spectrum is not intense.

### Palladium deposition

**Figure 7:**
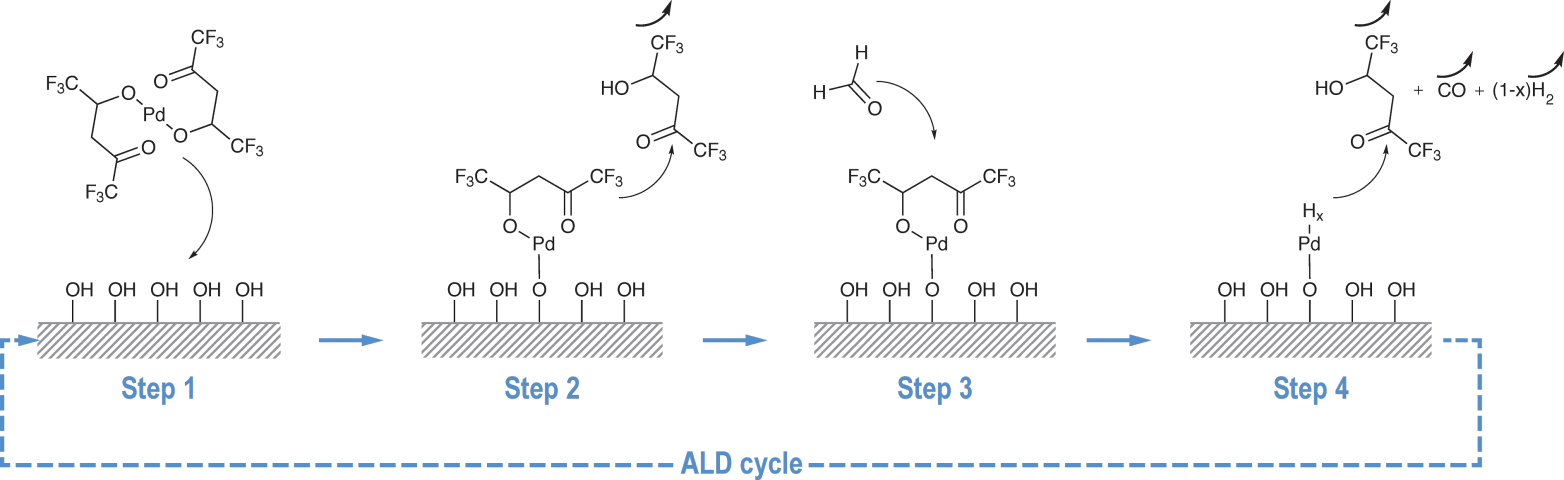
ALD sequence during Pd deposition from Pd(hfac)_2_ and formaldehyde.

A key advantage of ALD is that the growth of materials proceeds according to a two-dimensional mechanism. Nevertheless, for electrocatalytic applications, it is more suitable to have metallic clusters. To obtain such a morphology, it is possible to adjust the deposition parameters and the nature of the precursor. The outcome will depend also on the interaction between the substrate and the deposit. Recently, Elam et al. [25] have reported the synthesis of sub-nanometer Pd particles by an alternating exposure of the substrate to the metallic precursor and to trimethylaluminum. The active hydroxyl sites are thus occupied, which hinders the lateral growth of the particles. As mentioned in the introduction, the growth of Pd clusters by a direct ALD process, which uses palladium hexafluoroacetylacetone (Pd(hfac)_2_) and formaldehyde, has been previously described [25,36–38]. The mechanism is summarized in Figure 7. Steps 1 and 2 consist of the adsorption of the Pd(hfac)_2_ precursor onto the surface and its reaction with the hydroxyl sites and a subsequent H-hfac release. After the exposition of the surface to the second precursor (step 3), Pd(hfac) is reduced by formaldehyde. A –Pd–H_x_ termination is created at the active site and Hhfac, CO and H_2_ are released during step 4.

**Figure 8:**
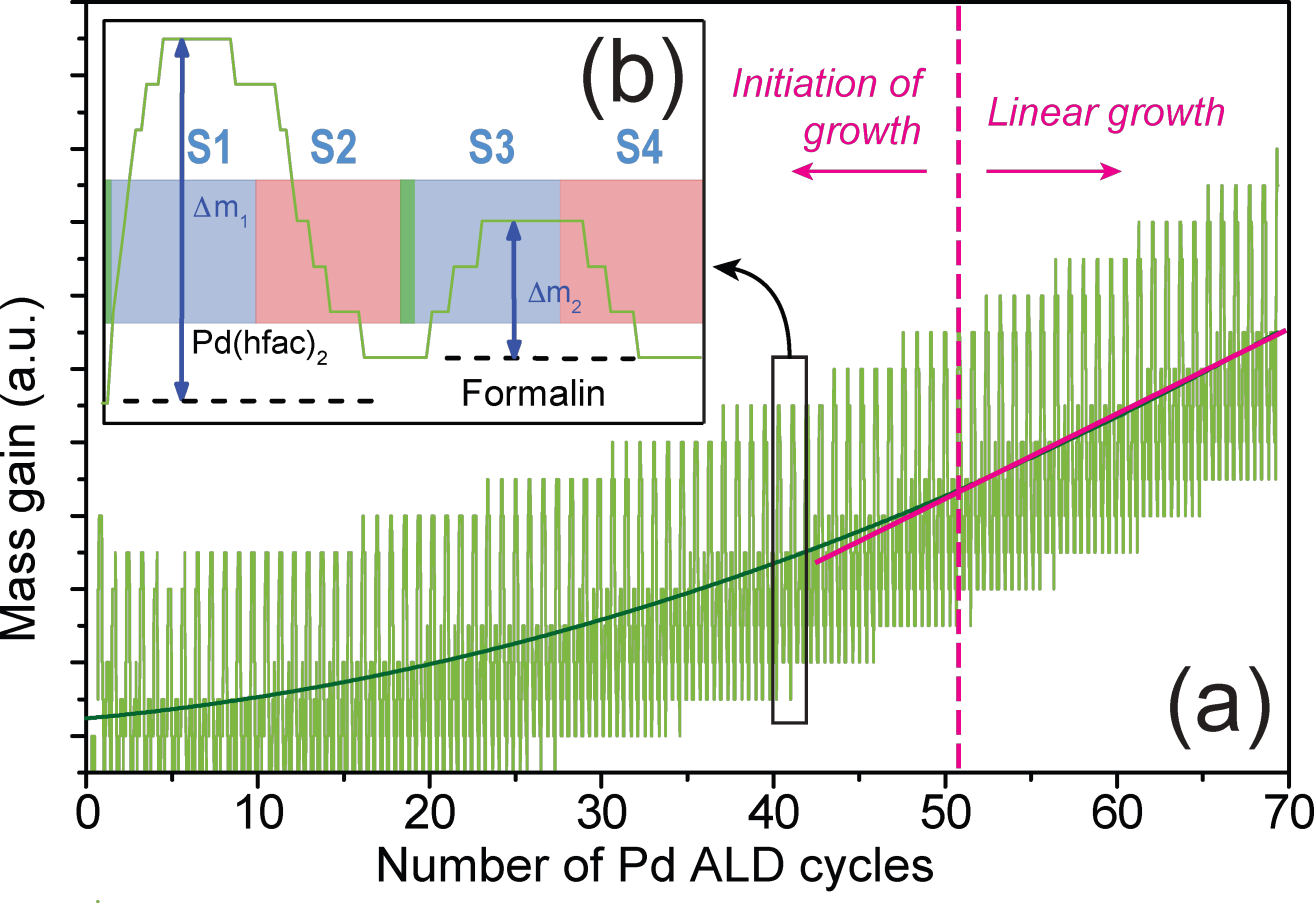
In situ QCM measurements of Pd mass gain during the ALD process for Pd. (a) General evolution and (b) enlarged view of one ALD cycle.

The formation process of the Pd clusters has been monitored by QCM measurements in order to detect the mass gain and loss during the ALD cycles. The general evolution of *m* during the deposition is shown in Figure 8a. Two growth regimes are identified in the curve: before and after 50 cycles. At first, the growth rate of Pd is low and non-linear. It progressively increases and reaches an almost linear growth after 50 ALD cycles. Such behavior has already been observed [36,39]. The initial low growth rate has been attributed to the long nucleation stage of the Pd clusters onto oxidized surfaces and/or ascribed to the surface poisoning by the precursor ligands by others [38]. An enlarged view on one ALD cycle (Figure 8b) shows the details of the mass gain and loss during the precursor pulses and the pumping of the exposition chamber. Step S1 consists of the adsorption of Pd(hfac)_2_ precursor molecules onto the surface. At this stage, the mass gain is denoted Δ*m*_1_. The end of the exposure time (S1) does not correspond exactly to the maximum of the gain mass Δ*m*_1_. The exposure time could thus be decreased to optimize the cycle duration. However, a long exposure duration assures the diffusion of the chemical species toward the pore tips. A similar observation can be done for the pumping time (step S2). Its duration is also not optimized but a longer purge and pumping stage would surely remove all the byproducts and the excess of reactants. George et al. [38] have shown that during the exposure with Pd(hfac)_2_, the released Hhfac can adsorb onto the hydroxylated Ni surface and block any further Pd reaction at those locations. As mentioned above, this phenomenon can hinder the lateral growth of the Pd film and slows down the deposition rate at the beginning. Steps S3 and S4 describe the surface exposure to the second precursor (formaldehyde) and the purge/pumping of the reactor, respectively. At the end of second part of the ALD cycle, the net mass variation should be negative. After the reaction of HCOH with –Ni–O–Pd(hfac), H-hfac, CO and H_2_ are indeed released. However, the QCM measurements show no mass loss. This unexpected measurement could again be attributed to the cooling effect of the vector gas on the quartz oscillation that has been mentioned above for Ni deposition. These data give only an indication on the general deposition process and cannot be used to interpret the growth mechanism. QCM data indicate that Pd deposition takes place onto the surface since the general trend is almost flat if no Pd is pulsed in the chamber.

**Figure 9:**
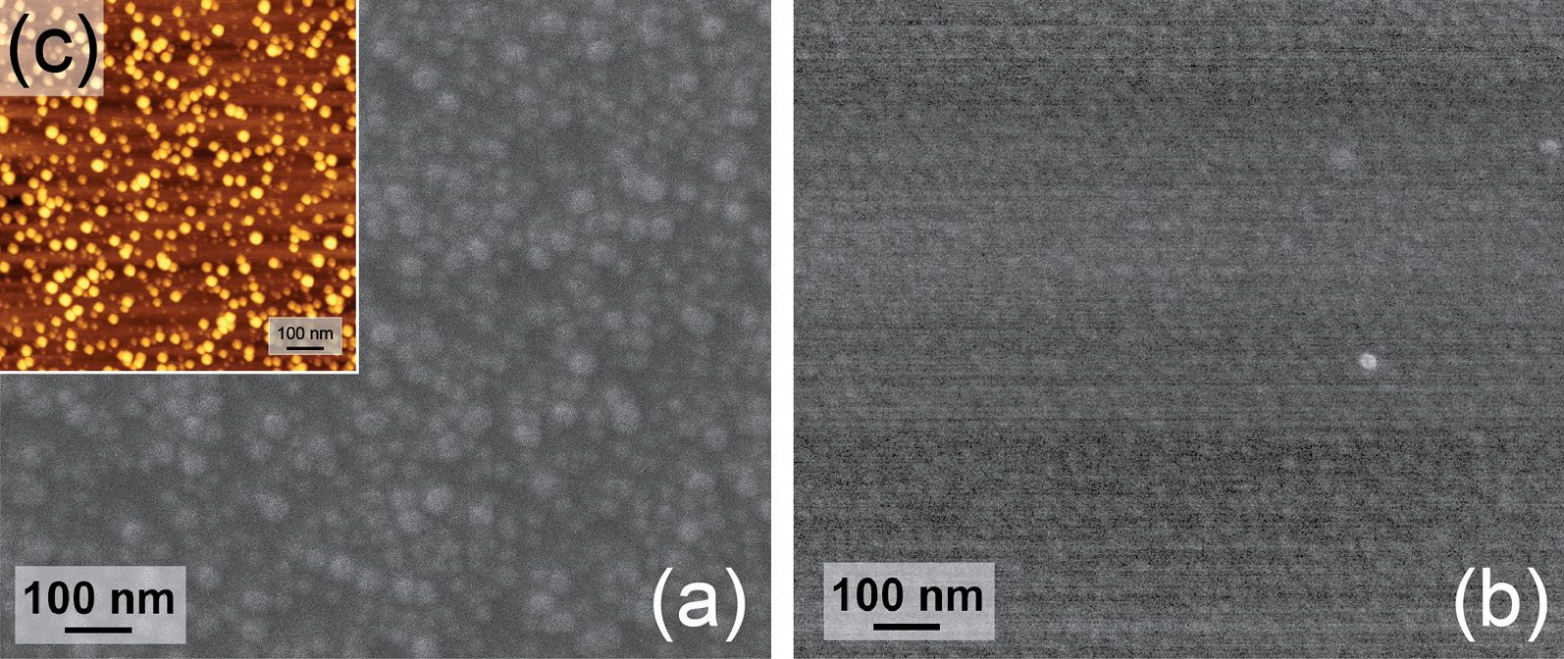
SEM top views of Pd deposits after 100 ALD cycles onto (a) as-grown NiO and (b) reduced NiO films on Si substrate. (c) AFM image of Pd clusters onto as-grown ALD NiO.

The morphologies of the Pd films grown onto the NiO layer have been observed by SEM and AFM with and without the reductive annealing treatment in H_2_. In order to facilitate such characterizations, the observed NiO and Pd layers have been grown onto flat Si substrates. Note that these depositions onto planar Si and onto AAO membranes have been performed simultaneously. Pd deposits carried out onto as-grown and annealed NiO layers that were grown before onto the Si wafers are presented in Figure 9a and Figure 9b, respectively. Their average diameters are, respectively, about 40 and 10–20 nm. The size of the clusters observed in Figure 9a cannot be attributed to the supporting NiO crystallites since their average size is in the range of 10–15 nm according to TEM cross section shown as supplemental material (Figure S2, Supporting Information File 1). The QCM, XPS and XRD measurements also attest the deposition of Pd onto the NiO layers. Such a spherical morphology suggests a Volmer–Weber growth mechanism of Pd. Such a formation of 3D islands is due to the high difference of surface energies between the metallic Pd and the oxidized support [39–40]. The formation of 3D islands can also be supported by the H-hfac ligands that are adsorbed on active –OH sites at the surface after the Pd(hfac)_2_ pulse [38]. In the case of Figure 9b, it was more difficult to observe the clusters with a high resolution. However the average size is slightly higher than the initial Ni/NiO layer. It indicates therefore that the Pd deposit covers the Ni/NiO film uniformly. This could be due to a lower surface energy between the Pd and the annealed substrate. It is even possible to form a Pd/Ni alloy if the NiO top layer appears only when the sample is exposed to air.

**Figure 10:**
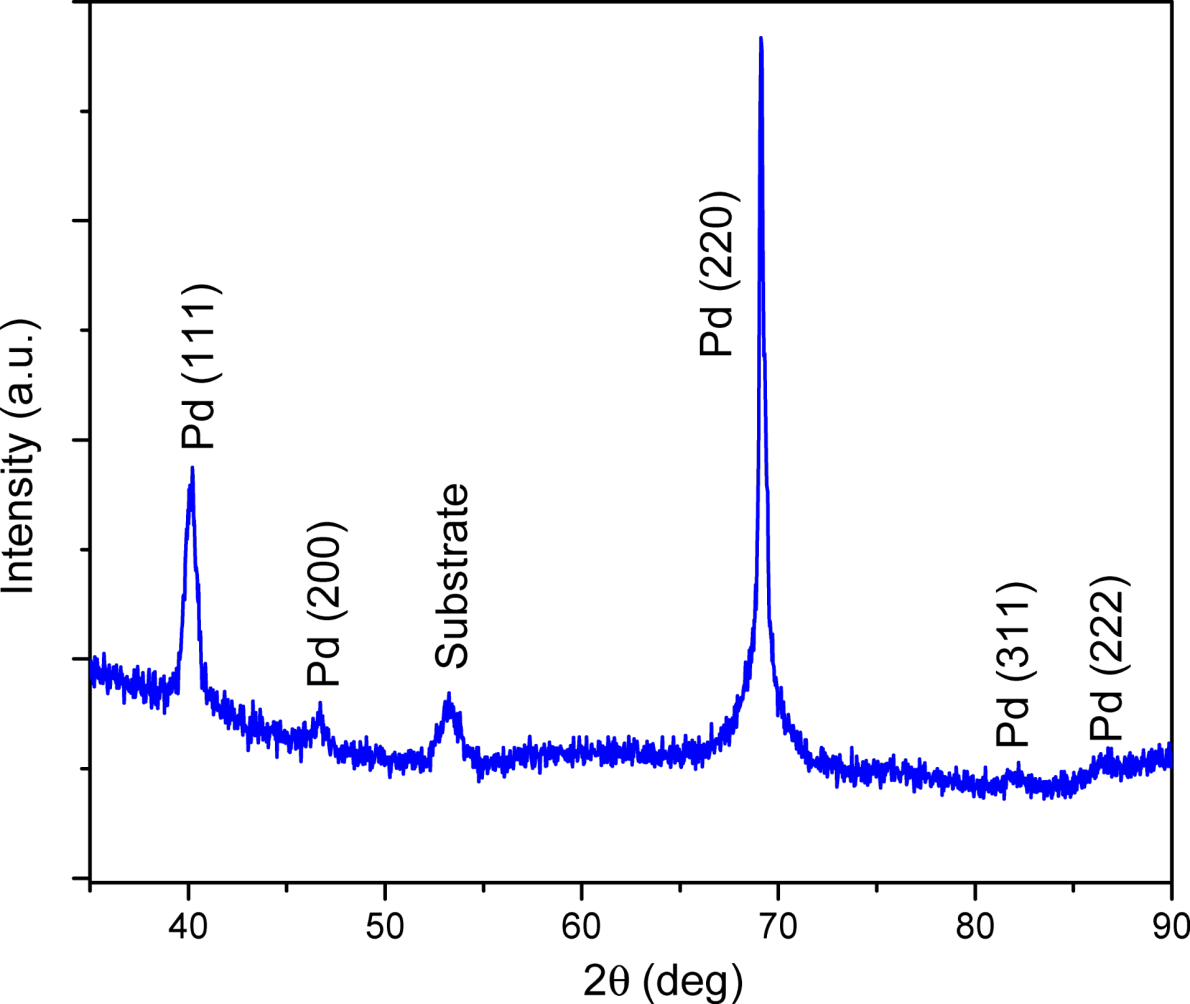
X-ray diffractogram of Pd deposited by ALD exhibiting a polycrystalline structure with a preferential orientation along the fcc [220] direction.

**Figure 11:**
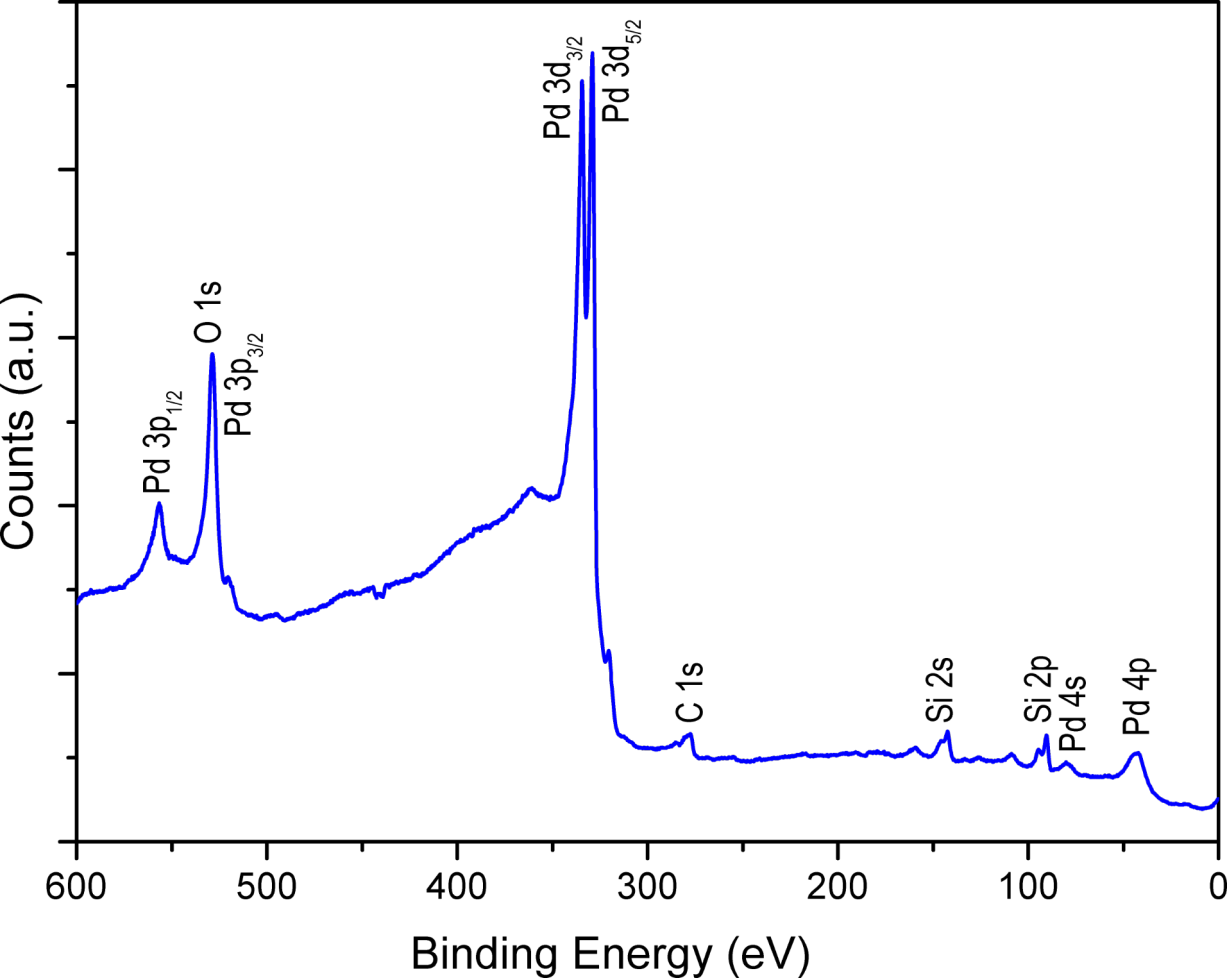
XPS survey spectrum of metallic Pd.

The two different growth mechanisms demonstrate the strong influence of the substrate on the deposition process. Although the XPS data indicate that the reduction of NiO to Ni is not total, the Pd deposition proceeds according to a 2D growth mechanism after the annealing in H_2_ atmosphere. In order to optimize the catalyst morphology, it appears that the Pd deposition should be performed onto as-grown NiO because bigger Pd islands are formed then. The AFM image presented in Figure 9c, shows clearly the Pd clusters that cover all of the Ni/NiO layer surface. Since Figure 9a and Figure 9b show planar substrates, they cannot be used to precisely evaluate the size of the Pd particles in the NiO/AAO system. However they give valuable information about the nucleation process of the Pd clusters on the NiO and Ni surfaces. Since atomic layer deposition is a self-limiting layer-by-layer process, it is reasonable to assume that the deposition occurs within the AAO/NiO structures but the particle size should be lower than the one observed on planar substrate. The crystal structure of Pd deposit has been investigated by X-ray diffraction. The XRD patterns shown in Figure 10 depict a polycrystalline structure of the Pd layer with a preferential orientation in the [220] direction (peak at about 70°). The Pd crystallographic structure is face centred cubic (fcc) similar to the structure of bulk Pd metal [11]. The XRD diffractogram suggests therefore the presence of metallic Pd. This result is further confirmed by the XPS analysis (Figure 11) that has revealed the presence of Pd, O and C on the surface. Similarly to Ni, the spectrum indicates the contribution of metallic and oxidized Pd with a slight contribution of O. The presence of Pd–O bonds can also be attributed to the oxidation occurring during the sample transfer to the XPS chamber or to the Pd deposition process itself. As observed for Ni, the Pd layer contains a small amount of C.

### Electrooxidation of HCOOH on Pd/Ni layers

**Figure 12:**
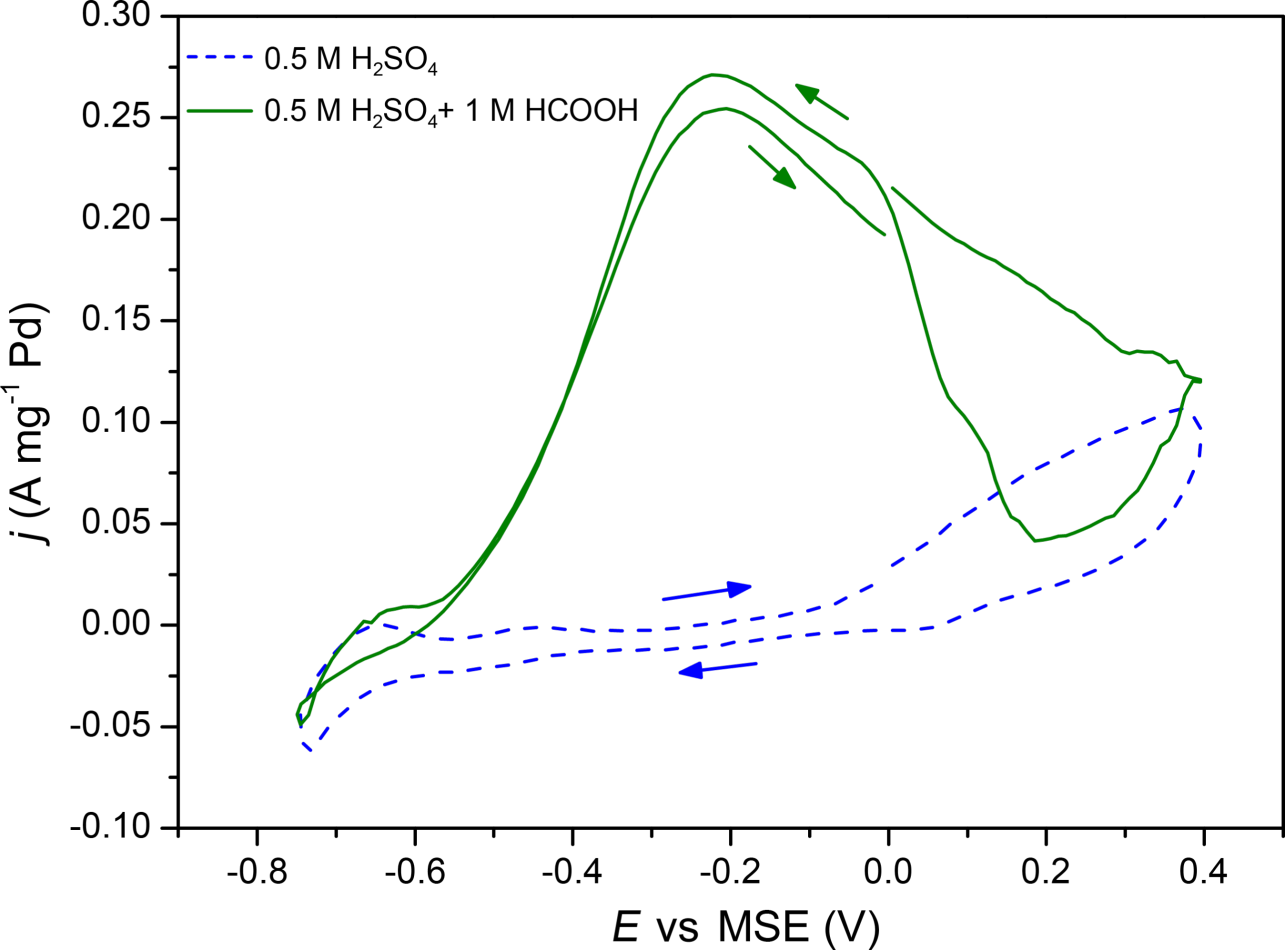
Cyclic voltammograms of Pd(100 ALD cycles)/Ni(1000 ALD cycles) catalysts in 0.5 M H_2_SO_4_ without (blue dashed line) and with (continuous green line) 1 M HCOOH at 15 mV·s^−1^. Current densities are given per unit mass of Pd estimated from the QCM measurements.

According to literature [10–13], Pd/Ni seems to be a more interesting system for the electrooxidation of HCOOH than Pd/NiO. The electrochemical characterizations have thus been performed with the H_2_-annealed NiO layers after the deposition of Pd. Figure 12 shows the forward and reverse scans of the third CV cycle on Pd/Ni electrocatalysts deposited onto an AAO membrane in 0.5 M H_2_SO_4_ before and after adding 1 M HCOOH. From cycle number 1 until cycle number 3, a decrease of the electrocatalytic activity of Pd/Ni system is observed. Indeed, the stability of Ni in H_2_SO_4_ is not as good as in KOH, however in the potential region of interest no Pd/Ni deactivation due to Ni dissolution has been observed. The third cycle is shown because after three cycles a stable and reproducible behavior of Pd/Ni system was obtained (CVs are identical) in the presence and absence of formic acid. The cyclic voltammogram disclosed in Figure S3 (Supporting Information File 1) shows the evolution of the current during the first six cycles. It demonstrates the stability of the Pd/Ni system over time in 0.5 M H_2_SO_4_.

**Figure 13:**
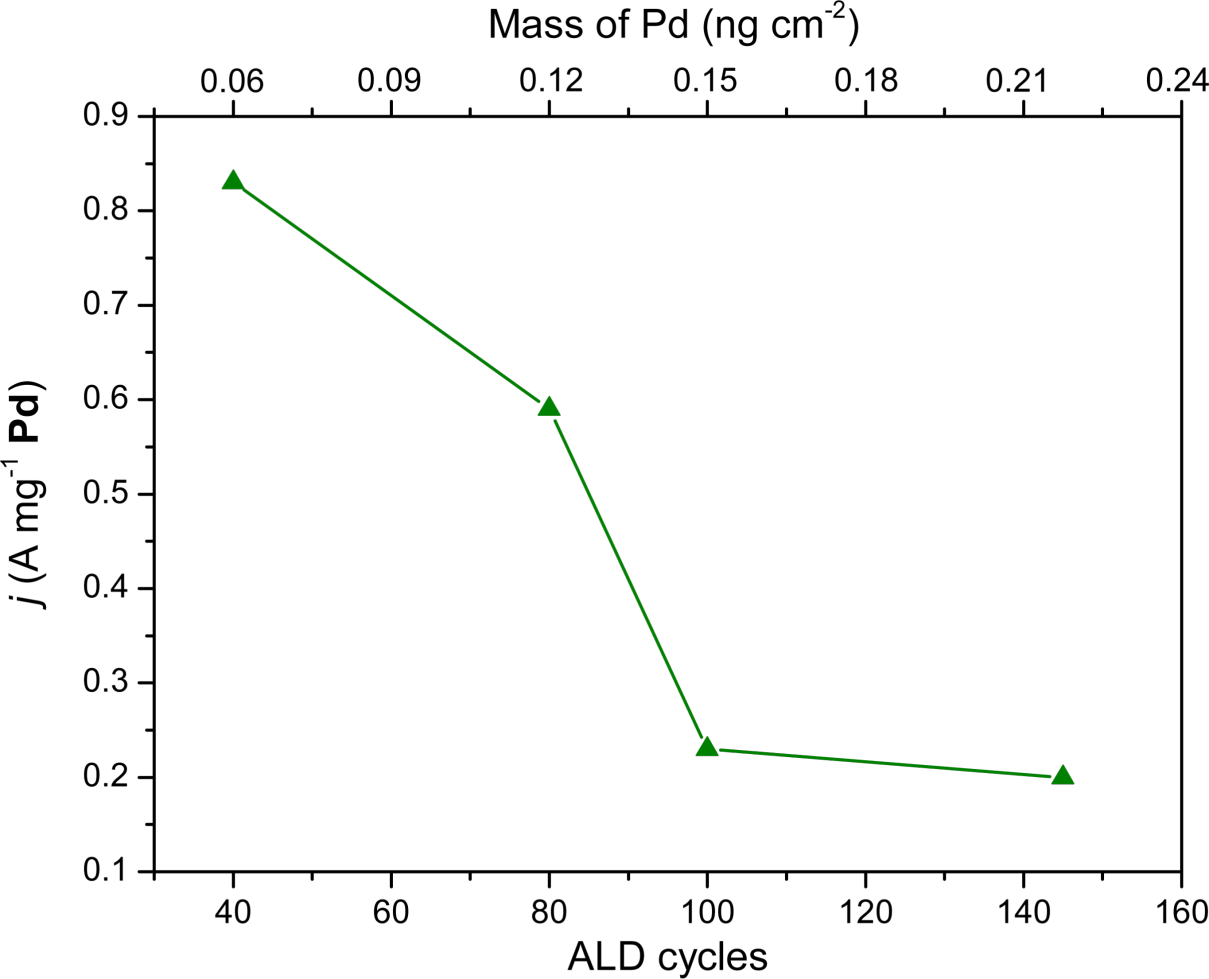
Peak current densities of the electrooxidation of 1 M HCOOH in 0.5 M H_2_SO_4_ with various Pd contents in the Pd/Ni nanocatalysts, which were obtained with 40 to 145 Pd ALD cycles. The mass of Pd was estimated from the QCM measurements.

The Ni and Pd layers were formed after 1000 and 100 ALD cycles, respectively, on alumina membranes. The applied potential varies from −0.75 to 0.4 V vs MSE at 15 mV·s^−1^. At potentials lower than −0.6 V, the H_2_ adsorption/desorption process is observed. Without formic acid in the solution, at potentials between −0.6 and 0 V, the voltammogram exhibits a flat region till approximately 0 V and at higher potentials the formation of Pd oxides starts to take place. In the presence of formic acid, on the other hand, the current begins to increase at a potential of −0.58 V reaching a maximum current density of 0.26 A·mg^−1^ at −0.2 V because of the oxidation of HCOOH. A further increase of the potential leads to a decrease of the current density due to the oxidation of the palladium and the inhibition of the catalytic activity of the metallic system by reaction intermediates [9,41]. On the reverse scan, the current remains low until 0.19 V, at which the reduction of PdO*_x_* begins to take place, and then increases because of the electrooxidation of formic acid on the reduced Pd. Note that the anodic wave that is centered at −0.19 V in the reverse scan is slightly higher than the one during the forward scan. This hysteresis indicates that the Pd surface still remains active and the previously formed oxides are completely reduced when the potential is reversed toward the negative direction. The electrooxidation of HCOOH follows the direct dehydrogenation pathway, which is in agreement with previous works [42]. The effect of the number of Pd ALD cycles (40, 80, 100 and 145) and, consequently, the Pd to Ni ratio on the current peak at −0.19 V, which corresponds to the oxidation of HCOOH is shown in Figure 13. Additionally, the electrochemical characterizations have shown the typical Pd response in H_2_SO_4_. The obtained results (overpotential for the oxidation of formic acid) are in a good agreement with the literature data reported for Pd/transition metal systems prepared by other techniques [10,13].

The mass activity of the Pd systems decreases to about one fourth from 40 to 145 ALD cycles. The increase of mass of Pd may result in the formation of larger clusters, and subsequently in both a smaller overall active surface area of the catalyst per unit mass, and weaker interactions with the Ni support. These two reasons can explain the negative trend of peak current densities for the oxidation of formic acid on Pd/Ni electrocatalysts with the increase of Pd mass. Note that it can also be because of the mass transport effect [43] since diffusion into such narrow channels can differ strongly from standard 2D models.

## Conclusion

In this study, well-defined Pd/Ni nanocatalysts grown by ALD have been investigated for the electrooxidation of formic acid in 0.5 M H_2_SO_4_. The deposition of nickel oxide from NiCp_2_ and O_3_ precursors on high aspect ratio nanoporous Al_2_O_3_ has been demonstrated. Although the chemical composition analysis of the NiO layers has not shown that the reductive treatment in H_2_ leads to fully metallic films, in which no strong morphological modifications were observed. Furthermore, it was concluded that the oxidized Ni is a better substrate to obtain a three-dimensional growth of Pd islands, which are more suitable for electrocatalytic applications. The Pd deposit is polycrystalline and exhibits a preferential orientation along the [220] direction. For both Ni and Pd depositions, the QCM results were not conclusive with regard to proper information about the deposition mechanisms by ALD. The Pd/Ni bimetallic systems demonstrates a high activity toward the electrooxidation reaction of formic acid and reaches 0.83 A·mg^−1^ for Pd(40 ALD cycles)/Ni(1000 ALD cycles). The electrochemical properties are very similar to those reported in the literature [10–13]. The interaction between Pd and Ni is stronger when the mass of the deposited Pd is decreased because of the lower thickness and size of Pd particles, but also because of the electronic effects between the alloyed Pd/Ni metals or because of the mass transport effect in 3D nanostructures. This explains the trend of higher peak current densities for the electrooxidation of formic acid at a lower Pd content in the Pd/Ni nanocatalysts.

## Experimental

**Figure 14:**
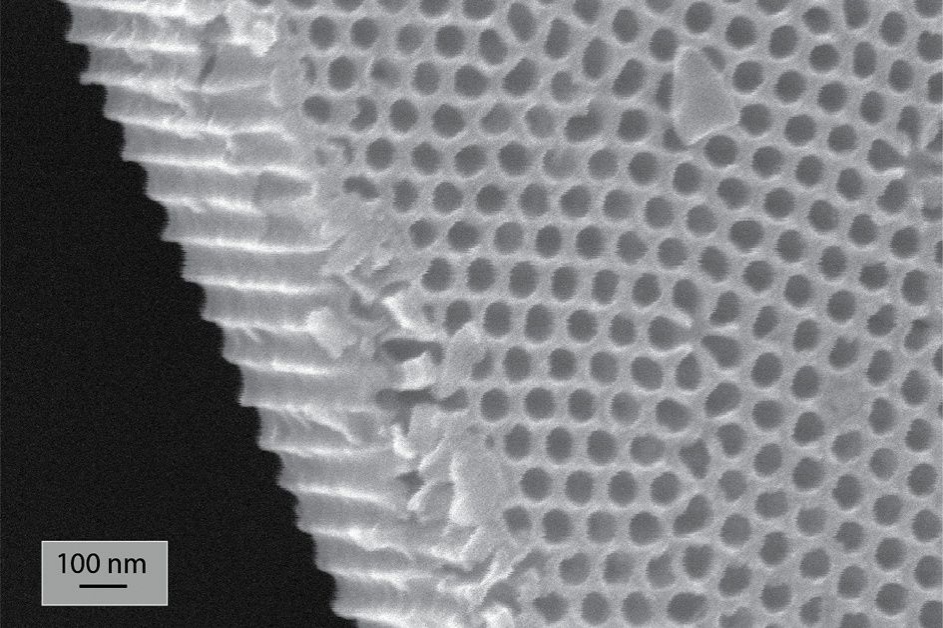
SEM micrograph of an anodic alumina oxide template. After the electropolishing, a sacrificial film was firstly grown by applying 40 V in 0.3 M H_2_C_2_O_4_ for 24 h at *T* = 8 °C and then dissolved in H_2_CrO_4_ and H_3_PO_4_ at 50 °C for 12 h. The second anodization step is then carried out during 2.5 h at the same anodic conditions.

The porous alumina structures have been grown on 4 cm wide aluminum discs (Goodfellow, 99.999%) by using the method that is schematically depicted in Figure 1a–e. The aluminum was first electropolished in an alcoholic solution of perchloric acid and successively anodized at a constant voltage, *U*, of 40 V in oxalic acid. A chemical dissolution was performed between the two anodizations to remove the disordered sacrificial Al_2_O_3_ layer. The resulting alumina membrane is ordered on a large scale area (ca. 10 cm^2^); the pore diameter is 40 nm and the length of the pores is about 5 μm. A typical AAO template is shown in Figure 14.

The Pd/Ni catalysts have been prepared by ALD in a Fiji 200 reactor from Ultratech/Cambridge Nanotech. The catalysts (Ni and Pd) were deposited both on AAO membranes and on flat Si(100) wafers that were cleaned beforehand by sonication in acetone, isopropanol and ethanol and deoxidized by dipping in 1% HF for 5 s. The alumina template was coated by a thin NiO layer grown from nickelocene (NiCp_2_, 99% from STREM chemicals), and ozone as chemical precursors. The temperature of the ALD chamber during the deposition was set to 250 °C. The ALD cycle consisted of successive exposures of the sample to NiCp_2_ and O_3_. The pulse durations and exposure time were, respectively, 1 and 30 s for NiCp_2_ and 0.2 and 20 s for O_3_. In both cases, the purging of the chamber was carried out for 30 s. The resulting NiO film was annealed and reduced under a H_2_ flow at 300 °C for 3 h to obtain a metallic Ni layer. The last step of the preparation of the catalysts consisted of depositing Pd nanoclusters that have been grown from palladium(II) hexafluoroacetylacetonate (Pd(hfac)_2_, 98% from STREM chemicals), and formalin (37% formaldehyde in water with 10–15% of methanol from Sigma-Aldrich) at 200 °C. The ALD sequence consisted of successive exposures of the Ni-covered sample to Pd(hfac)_2_ and formaldehyde. The pulse durations were, respectively, 1 and 3 s. In both cases, the exposure and purge durations were 30 s. Due to the low vapor pressure of the Pd precursor, an argon flow has been injected in the canister for 0.25 s through an additional valve before each precursor pulse in order to enhance the transport of chemical species toward the deposition chamber. Experiments with various number of ALD cycles of the different precursors have been performed to adjust the mass and the composition of the films. A chemical etching performed in chromic acid solution allows to chemically dissolve the alumina template to allow for the nanostructured catalysts to be collected by centrifugation for further TEM observations of the catalysts out of the alumina template. The Pd/Ni catalysts have been deposited both on 3D alumina templates and flat Si(100) wafers in order to facilitate the chemical and structural characterizations. In situ monitoring of the relative mass gain and loss was performed by using a quartz crystal microbalance (QCM from Inficon). The QCM is connected to the ALD chamber and driven by a SQM-160 controller for data acquisition. The morphology of Al_2_O_3_ templates and Pd/Ni electrocatalysts has been observed by SEM and TEM using, respectively, JEOL 6320-F and JEOL 3010 equipment. Some additional morphological investigations have been carried out by non-contact AFM using a XE 100 microscope from Park systems. The crystalline structure of NiO, Ni and Pd has been characterized by X-ray diffraction using an INEL diffractometer equipped with a quartz monochromator and a horizontally disposed 1D curved position detector (CPS-120) that covers a 2θ angle of 120°. The measurements were obtained in reflection mode with an incident angle of 10° and Cu Kα1 (1.54056 Å) radiation. X-ray photoelectron spectroscopy by using a Mg electrode Kα (1253.6 eV) source (HA150 from VSW) was used for surface chemistry composition analysis. The Electrooxidation of HCOOH on alumina-supported Pd/Ni catalysts after reductive annealing treatment of the NiO ALD layer has been studied in 0.5 M H_2_SO_4_ solution in a three-electrode teflon cell. A large surface area Pt mesh and a mercury sulfate electrode (MSE) served respectively as counter and reference electrodes. The geometric area of the working electrode was 0.196 cm^2^. The electrical contact to the working electrode was established by a gold wire on the Pd/Ni layer. Cyclic voltammetry was carried out by using a BioLogic VSP potentiostat together with the EC-Lab software at room temperature. The CVs were performed in the potential region from −0.75 to 0.4 V vs MSE at a scanning rate of 15 mV·s^−1^. The current densities have been reported per unit mass of Pd (details on the Pd mass calculations can be found in Supporting Information File 1).

## Supporting Information

File 1 Additional experimental details
