# Supplementary material for: 3D-nanoarchitectured Pd/Ni catalysts prepared by atomic layer deposition for the electrooxidation of formic acid
Source: Beilstein J Nanotechnol. 2014 Feb 12;5:162–72. doi: 10.3762/bjnano.5.16 (PMC3943891; doi:10.3762/bjnano.5.16)
Supplement: File 1 — Additional experimental details [file Beilstein_J_Nanotechnol-05-162-s001.pdf]

Supporting Information

for

3D-nanoarchitectured Pd/Ni catalysts prepared by atomic layer deposition for the electrooxidation of formic acid

Loïc Assaud[1], Evans Monyoncho[2], Kristina Pitzschel[1], Anis Allagui[2], Matthieu Petit[1], Margrit Hanbücken[1], Elena A. Baranova*[2] and Lionel Santinacci*[1]

Address: [1]Aix-Marseille Université, CNRS, CINaM UMR 7325, 13288, Marseille, France and [2]Department of Chemical and Biological Engineering, Center for Catalysis Research and Innova- tion, University of Ottawa, 161 Louis-Pasteur St., Ottawa, ON, K1N 6N5, Canada

Email: Elena A. Baranova - elena.baranova@uottawa.ca;

Lionel Santinacci - lionel.santinacci@univ-amu.fr

* Corresponding author

Additional experimental details

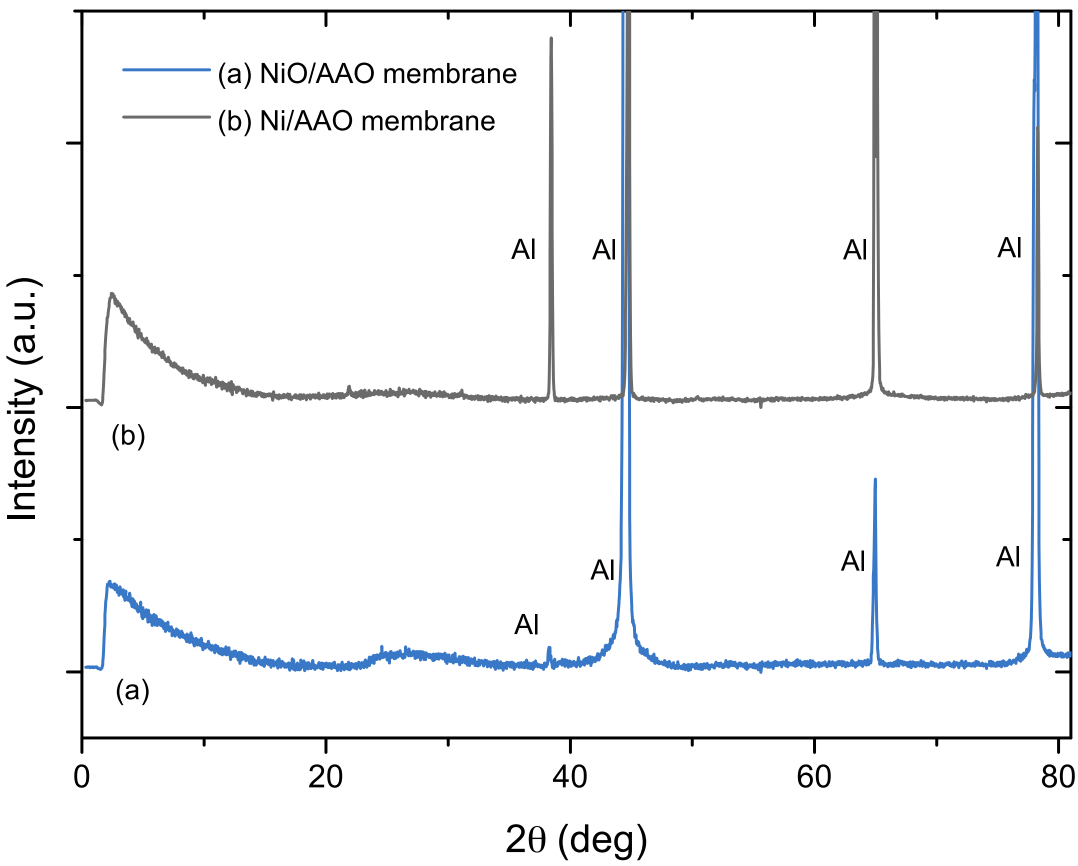

Figure S1: XRD diffractogram showing the amorphous structure of NiO deposited on AAO membrane before (a) and after (b) reductive annealing in H_2.

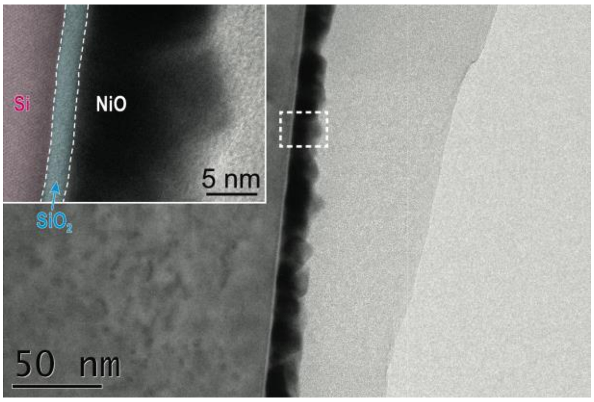

Figure S2: TEM cross-section view of a continuous NiO layer deposited by ALD on flat Si (100) surface. A SiO_2 layer is observed at the interface.

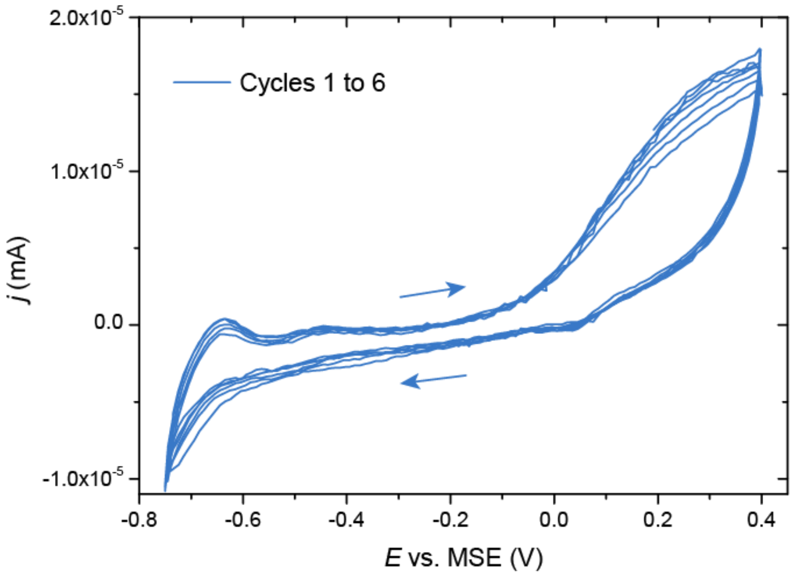

Figure S3: Six successive cyclic voltammograms showing the electrochemical activity of 3D Pd/Ni catalysts in 0.5 M H_2SO_4 (the number of Pd ALD cycles is 100). The current-vs-potential curves are quite reproducible over the time.

Detailed calculation of the deposited mass of Pd

To determine the mass per unit surface of deposited Pd by ALD, a geometric estimation of the distribution of catalysts on alumina templates has been calculated as follows. The Ni geometry has been considered to be a continuous layer covering the whole inside surface of alumina pores. This approximation is quite realistic with reference to the SEM image of Ni shown in Figure 5.

The mass per unit surface of Pd can be subsequently determined by considering these assumptions and knowing the pore density of hexagonal distribution of cells on the alumina support that is given by the following equation [1]:

$$n = \frac{2\,.10^{14}}{\sqrt{3}.D_{int}^{2}} \qquad (1)$$

where D_{int} denotes the pitch between two consecutive pores determined empirically by $D_{int} = \lambda_C \cdot U$ with $\lambda_C = 2.5$ being a constant of proportionality in nm·V^{-1} and U being the applied voltage during the anodization step.

The pore diameter is 40 nm and the deposited Ni on the alumina template has a thickness of about 10 nm according to TEM and SEM observations (Figures 4 and 5), then the diameter of alumina pores is subsequently reduced to 20 nm. Equation 1 gives a number of pores of $2.27 \cdot 10^9$ in the exposed surface area of the used working electrode for the electrochemical measurements. The mass per unit surface of Pd has been calculated by using these approximations for each variation of number of ALD cycles (with a growth per cycle of 0.2 Å/cycle according to [2]).

References

1. Sulka, G. D.; Highly Ordered Anodic Porous Alumina Formation by Self-Organized Anodizing. In *Nanostructured Materials in Electrochemistry*; Eftekhari, A., Ed.;Wiley-VCH: Weinheim, 2008; pp 1–116.

2. Martinson, A. B. F.; DeVries, M. J.; Libera, J. A.; Christensen, S. T.; Hupp, J. T.; Pellin, M. J.; Elam, J. W. *J. Phys. Chem. C* **2011,** *115,* 4333–4339. doi:10.1021/jp110203x
